# Enzymatic and Non-Enzymatic Molecules with Antioxidant Function

**DOI:** 10.3390/antiox10040579

**Published:** 2021-04-09

**Authors:** Paola Irato, Gianfranco Santovito

**Affiliations:** Department of Biology, University of Padova, 35131 Padova, Italy; gianfranco.santovito@unipd.it

It is well known that the excessive production of reactive oxygen species (ROS) can lead to the peroxidation of membrane lipids, glycation/oxidation/nitration of proteins, inactivation of enzymes, DNA mutation and damage, and other alterations in the subcellular components [1,2]. Faulty damage repair can lead to oxidative stress increasing the probability of cell death or promote cancer through the accumulation of DNA mutations. But it is also known that living organisms (at all levels of complexity) evolved antioxidant molecules and proteins to prevent or repair oxidative damages [3,4,5,6,7].

The Special Issue “Enzymatic and Non-Enzymatic Molecules with Antioxidant Function” has been conceived to implement the knowledge about antioxidant molecules in microorganisms, plants, and animals. Here, we offer an overview of the contents of this Special Issue, which collects 13 original articles and three reviews. In particular, both enzymatic (alpha-dioxygenase, ascorbate peroxidase, catalase, dehydroascorbate reductases, glutathione peroxidase, glutathione reductase, glutathione-S-transferase, NADPH oxidase, peroxiredoxin, and superoxide dismutase) and non-enzymatic molecules (ascorbate, fibrin, glutathione, melatonin, mycothiol, phenolics, and serum albumin) were studied in a wide range of scientific contexts (biotechnology, crop production, developmental biology, ecotoxicology, evolution, human health).

Catalase (CAT), glutathione peroxidase (GPx), and superoxide dismutase (SOD) are functionally interconnected because the product of the reaction catalyzed by SOD, hydrogen peroxide (H_2_O_2_), is the substrate of both CAT and GPx. It is, therefore, interesting to compare the molecular evolution of corresponding gene families. The work of Chovanová et al. focused on the genomes of thermophilic fungi: SOD genes remain constant during long-term evolution, while CAT genes are reduced in comparison with their mesophilic counterparts [8]. These results have important implications for the physiology of ROS metabolism in eukaryotic cells at elevated temperatures. The study of Ferro et al. analyzes the evolutionary relationships among the GPxs of the unicellular eukaryotic model organism *Tetrahymena thermophila* and the orthologous enzymes of phylogenetically related species [9]. In particular, results highlighted the conservation of transcriptional regulatory elements in the promoter region of GPx genes, suggesting a tight control of specialized signaling pathways. Li et al. studied Eucalyptus, a plant that is subjected to various biotic and abiotic stresses and that evolved antioxidant gene families characterized by a number of multiple isoforms higher than most of other angiosperms, giving higher adaptability to environmental changes and stresses [10]. These results will help better understand the genetic differences between closely related species and stimulate additional studies on the mechanisms that underlie speciation and biodiversification.

The results of Loi et al. proposed an ascorbate–glutathione regulation network under oxidative stress and may represent a new way to increase the plant antioxidant defense system, plant nutraceutical value, and the health benefits of plant consumption [11]. Dehydroascorbate reductases (DHARs) are important enzymes involved in the plant response to oxidative stress, such as that induced by the mycotoxin beauvericin (BEA). In this study, tomato plants were treated with BEA and DHARs, and dehydroascorbic acid (DHA) and glutathione (GSH) were determined. In addition, Hayat et al. utilized tomato to study the effects of aqueous garlic extracts (AGEs), diallyl disulfide, and allicin during the seed-to-seedling transition of plants. Seeds germinate with AGE exhibit induced defense via antioxidant enzyme activities [12]. The tomato has also been used as a model organism by Wang et al. to study the role of melatonin as an antioxidant agent [13]. The authors generated melatonin-deficient and melatonin-rich tomato plants, and they found that melatonin deficiency increased the production of ROS and impaired antioxidant capacity.

Arsenic (As), as well as other chemical elements, contaminates the food chain and decreases agricultural production through impairing plants due to oxidative stress. Kofroňová et al. investigated As tolerance mechanisms utilizing tobacco plants As-sensitive and As-tolerant. The total antioxidant capacity was far stronger in the As-tolerant genotype, showing higher antioxidants levels (phenolics, ascorbate, GSH) [14]. Moreover, malondialdehyde levels, a lipid peroxidation marker, increased only in As-sensitive plants.

Furthermore, exposure to metal ions can increase the ROS formation rate. Muszyńska et al. studied Silene vulgaris ecotypes with different levels of metal tolerance in relation to mechanisms involved in protection against the imbalanced generation of ROS after treatment with Zn, Pb, and Cd [15]. Their findings confirmed the sensitivity of the non-metallicolous ecotype, provide a better understanding of the operation of antioxidant machinery in metallicolous and non-metal tolerant specimens, and place peroxidases among the best antioxidants.

The toxic action of Cd can also manifest itself in the oral cavity through damage related to oxidative stress induced by this metal. The study of Dąbrowski et al. investigated whether the administration of an extract from *Aronia melanocarpa* berries, characterized by their strong antioxidative potential, may have a beneficial impact on the oxidative–reductive status of the submandibular gland under low-level and moderate human environmental exposure to Cd [16]. They evaluated the main antioxidant biomarkers, such as glutathione reductase, SOD, CAT, GSH, total antioxidative status (TAS), total oxidative status (TOS), oxidative stress index (TOS/TAS), and lipid peroxidation, as well as Cd accumulation. The results demonstrated that *Aronia* has a beneficial impact on the oxidative–reductive status, preventing oxidative stress development in salivary glands.

In another study related to the oral cavity, Kargarpour et al. exposed gingival fibroblasts to H_2_O_2_ with and without lysates obtained from platelet-rich fibrin (PRF) membranes, platelet-poor plasma (PPP), heated PPP, and the buffy coat [17]. The aim was to verify if H_2_O_2_ toxicity can be neutralized, and thereby local oxidative stress can be counteracted. They concluded that PRF, PPP, and the buffy coat can neutralize H_2_O_2_ through the release of heat-sensitive CAT.

Microorganisms also have powerful antioxidant systems, enzymatic and non-enzymatic, that become important in aerobic bioreactor cultivations. *Corynebacterium glutamicum*, a widely used industrial platform organism, uses mycothiol (MSH) as a major thiol non-enzymatic antioxidant. Hartmann et al. studied the role of MSH in this bacterium at different oxygen levels and concluded that MSH is an essential antioxidant to maintain the robustness and industrial performance of *C. glutamicum* during aerobic fermentation processes [18].

Muhtadi et al. investigated, in the yeast *Schizosaccharomyces pombe*, the role of CAT and the Fe^2+^/Mn^2+^ symporter in protecting meiotic chromosome dynamic and gamete formation from radicals generated by ROS and ionizing radiation [19].

Diabetes is a global endemic disease with rapidly increasing prevalence in both developing and developed countries. Chronic hyperglycemia generates oxidative stress in pancreatic β-cells, which are particularly vulnerable to the damaging effects of producing excessive ROS. Many studies suggest that increased oxidative stress and changes in lipid metabolism are involved in the pathogenesis and progression of diabetic tissue damage. In this context, Sierra-Campos et al. studied the effects of *Moringa oleifera* leaf extract on diabetic rats and they concluded that this extract is able to influence the catalytic activities of paraoxonase 1 and CAT to compensate for the changes provoked by diabetes in rats [20].

The review by Belinskaia et al. evaluated data published in the literature on the mechanisms of the enzymatic and non-enzymatic activities of albumin that determine its participation in redox modulation of plasma and intercellular fluid [21]. Blood is exposed to oxidants to a greater extent than the intracellular environment, and serum albumin plays a key role in antioxidant defense. The albumin molecule contains 17 disulfide bonds and one free thiol group in Cys^34^. The latter largely determines the participation of albumin in redox reactions. The number of disulfide bonds and Cys^34^ are conserved in all types of albumin. The Cys^34^ residue can neutralize ROS and reactive nitrogen species, such as H_2_O_2_, peroxynitrite, superoxide anion, and hypochlorous acid, being oxidized to sulfenic acid. The binding of some compounds affects the reactivity of the thiol group of Cys^34^ and modulates the antioxidant properties of the protein in the direction of strengthening or weakening. The properties of albumin should be considered in the development of therapy for pathologies associated with oxidative stress.

The review by Xie et al. summarizes data from several studies that have demonstrated how chronic obesity results in a state of systemic inflammation that has many downstream effects, increased ROS production included [22]. These effects are further exacerbated by SARS-CoV-2, which induces a cytokine storm, with an increased risk of renal dysfunction and subsequent heart failure in COVID-19 patients with obesity. This review also described the potential antioxidant drugs and the role of NaKtide (and their demonstrated antioxidant effect) used as a major strategy in the context of the COVID pandemic.

Finally, the last review by Boyd et al. explored the routes of Cu delivery that are utilized to activate cytoplasmic SOD and the usefulness and necessity of each [23]. Cu is tightly regulated due to the vital roles it plays within the cell but also because of its potential for adverse redox activity. Cu regulation is important to cellular function, and dysfunction often leads to disease, so therapeutics targeting Cu maintenance could be useful in treatment.
